# Symptoms of older orthopedic and rheumatic patients: A telephone survey about symptoms, symptom communication, treatment and further support after hospital discharge

**DOI:** 10.1007/s00391-022-02155-y

**Published:** 2023-01-24

**Authors:** Susanne Schiek, Katharina Hintzer, Carolin Dahley, Kathrin Wernecke, Birgit Feindt, Christoph Baerwald, Ulrich J. A. Spiegl, Thilo Bertsche

**Affiliations:** 1 Department of Clinical Pharmacy, Institute of Pharmacy, Leipzig University, Bruederstraße 32, 04103 Leipzig, Germany; 2 Drug Safety Center, University Hospital Leipzig and Leipzig University, Leipzig, Germany; 3 Central Patient Management, University Hospital Leipzig, Leipzig, Germany; 4 Department of Rheumatology, University Hospital Leipzig, Leipzig, Germany; 5 Department of Orthopaedics, Trauma Surgery and Reconstructive Surgery, University of Leipzig, Leipzig, Germany

**Keywords:** Geriatrics, Patient discharge, Symptom assessment, Orthopedics, Multimorbidity, Geriatrische Patienten, Entlassung, Symptom-Assessment, Orthopädie, Multimorbidität

## Abstract

**Background:**

In older multimorbid orthopedic and rheumatic patients, data on symptoms besides pain or reduced mobility are rarely published.

**Objective:**

We investigated patients’ perspectives on their symptoms after hospital discharge.

**Material and methods:**

Orthopedic and rheumatic patients aged over 70 years were asked via telephone interviews about (i) their symptoms, (ii) communication, (iii) treatment, and (iv) support.

**Results:**

(i) The 60 participants (35 women and 25 men) reported a median of 6 (min-max: 1–14) different symptoms, of which 86% (356 of 415) had existed before hospitalization, (ii) patients did not communicate 28% (117) of symptoms to either healthcare professionals, family or friends and (iii) 52 (87%) patients desired improvement. Of the 280 most impairing symptoms, 19% (52) were not treated at all. (iv) Almost all patients (59; 98%) considered it easy to obtain support.

**Conclusion:**

Remarkably, many symptoms were not communicated or treated despite the patients having been hospitalized.

**Supplementary Information:**

The online version of this article (10.1007/s00391-022-02155-y) contains supplementary material, which is available to authorized users.

## Abstract (de)

**Hintergrund:**

Bei multimorbiden orthopädischen und rheumatischen Patienten ist wenig über Symptome außer Schmerzen und Bewegungseinschränkung bekannt.

**Ziel:**

Es wurde die Perspektive der Patienten bezüglich ihrer Symptome nach stationärer Entlassung untersucht.

**Material und Methoden:**

Mittels Telefoninterviews wurden orthopädische und rheumatische Patienten über 70 Jahre zu (i) ihren Symptomen befragt. Zudem wurden (ii) Kommunikation, (iii) Behandlung und (iv) Unterstützung erfasst.

**Ergebnisse:**

(i) Die 60 Patienten (35 Frauen und 25 Männer) berichteten im Median 6 (min-max: 1–14) unterschiedliche Symptome, von denen 86 % (356 von 415) bereits vor Hospitalisierung existierten. (ii) Patienten kommunizierten 28 % (117) ihrer Symptome bisher weder dem Pflegepersonal noch ihrer Familie oder Freunden, (iii) 52 Patienten (87 %) wünschten sich eine Verbesserung. Von den 280 am stärksten beeinträchtigenden Symptomen wurden 19 % (52) nicht behandelt. (iv) Fast alle Patienten (59; 98 %) fanden es leicht, Unterstützung zu erhalten.

**Diskussion:**

Es überrascht, dass eine beachtliche Anzahl an Symptomen nicht kommuniziert oder behandelt wurde, obwohl die Patienten zuvor hospitalisiert worden waren.

## Brief introduction to the subject

Older patients suffer from various symptoms. This can be especially assumed for older orthopedic and rheumatic inpatients; however, it is unclear whether gaps in symptom management exist in routine care. We interviewed these patients after hospital discharge and aimed to investigate their symptoms, including those they had not reported so far. We further asked the patients what they suspected was the cause of their symptoms, how they treated them, and how they rated the support. This study supports raising awareness among healthcare professionals regarding the patients’ perspectives.

## Introduction and background

Many orthopedic or rheumatic patients are of higher age with multimorbidity. These patients often have to cope with pain [16] or mobility limitations [21]. Older multimorbid patients, in general, suffer from a high symptom burden [7]. Symptoms are subjective physical or mental experiences that indicate a bodily change in normal functioning [6, 18]. This often negatively influences the quality of life [23] or healthcare costs [14] and is, therefore, of high clinical relevance. Symptom experience is multidimensional and requires complex management strategies [18].

Nevertheless, limited data exist in orthopedic and rheumatic patients about other geriatric symptoms. The period after hospital discharge is of interest for two reasons. First, it is a critical time frame for older patients. Difficulties with follow-up appointments and medication [2] or transferring from an acute hospital to ambulatory care can worsen their general condition [4]. This might result in additional symptoms. Second, hospitalization and discharge represent a time frame during which the patient is in contact with multiple healthcare professionals. In the hospital setting, the patients see physicians of the specialized ward, who provide (symptom) diagnosis and treatment. Thereby treatment of symptoms can also mean a reduction of treatment (e.g., deprescribing). After discharge, the general practitioner (GP) usually manages the initiated (drug) treatment. In the community pharmacy, patients receive newly prescribed drugs and are counseled on how to administer them correctly. Thus, one might expect the chance of detecting patients’ symptoms is relatively high during this time period; however, it is not known to what extent patients themselves actively ask the involved professionals for support in the symptom management or whether barriers hinder patients from obtaining support. Findings on this could help to optimize the resources of interdisciplinary care.

We therefore aimed to analyze postdischarge symptoms of older multimorbid orthopedic and rheumatic patients. We focused on two patient groups with an assumed high number of symptoms to understand whether gaps exist in symptom management. We intended to explore the basis of the patients’ experienced symptoms, to what causes the patients attributed their symptoms, whether they had communicated these symptoms before, what measures they took to treat them, and whether they obtained further support to cope with them.

## Study design and investigation methods

### Ethics

The study has been approved by the appropriate ethics committees. Informed consent was obtained from all patients included in the study. Additionally, the respective responsible physicians gave their written consent to study participation.

### Study design

We performed a cross-sectional study by using a semi-structured telephone interview.

### Participants and setting

The participants were recruited in a university hospital’s three orthopedic wards (treatment focus on endoprosthetics, reconstructive joint interventions, and general traumatology) and one rheumatological ward (treatment focus on rheumatic diseases). Older patients admitted to one of these wards were invited to participate during their hospital stay. Inclusion criteria were age at least 70 years old (to enrol older patients), living independently, taking at least 5 drugs (to enrol patients with polymedication to treat multimorbidity), sufficient cognition assessed by the hospital physician, and written informed consent. Additionally, the patients needed to be able to talk on the telephone (adequate hearing and language skills).

### Development of the semi-structured interview

We used a quantitative explorative semi-structured telephone interview to assess the extent and variety of symptoms including those that might not have been communicated before. This design should facilitate the participation of older multimorbid patients in a telephone survey.

Two pharmacists with experience in palliative and geriatric pharmacy developed a semi-structured interview based on Somers et al. [22], the Memorial Symptom Assessment Scale [17] and the Common Terminology for Adverse Events [15]. Physicians with experience in geriatrics were involved for consultation.

To ensure comprehensibility and feasibility, the interview guide was cognitively pretested (by paraphrasing and comprehension probing) stepwise with nine professionals (pharmacists not included in the study design) and with three laypersons (older patients within the inclusion criteria). The participants in the pretest were not involved in developing the study protocol or the main study. The results of the pretests were not included in the final data assessment. The pretest with the professionals resulted in improved comprehensibility and notes to adjust the interview duration. The lay pretest resulted in the following modifications: inclusion of the symptom “hearing impaired”, shortening the interview guide (not asking which activities of daily living were impaired by the symptom, asking about treatment for only the 5 most severe or impairing symptoms), simplifying the wording in questions to increase lay understanding, including the response categories “ward physician” and “nobody” for the question about who recommended treatment.

The final interview (Supplement 1) consisted of four parts: (i) symptom characteristics (occurrence, intensity, impairment, timing, and cause), (ii) symptom communication, (iii) symptom treatment, and (iv) further support.

#### (i) Symptom characteristics

The interviewer discussed 24 predefined geriatric symptoms one after the other and asked for each whether the patient currently experienced the symptom (occurrence), how severe the symptom was (intensity: severe, moderate, weak, fully treated, not specified), how much it impaired them in their everyday life (impairment: severe, moderate, weak, none, not specified) and when the symptom first occurred (timing: before hospitalization, during hospitalization or after discharge). The patients were further asked what they considered the main causes of their symptoms. Age, disease and drug treatment were predefined categorized answers. Other named causes were also documented.

#### (ii) Symptom communication

The patients were asked with whom they had discussed the symptom before (physician, pharmacist, others, or nobody). In cases where the patients explained this in more detail, causes for the lack of communication were documented.

#### (iii) Symptom treatment

We asked the patients what measures they already took to improve the symptoms (drug and non-drug measures, asked for the five most severe or impairing symptoms), how effective these measures were (good, fair, poor) and who recommended the treatment (GP, specialist, ward physician, pharmacy, nursing service, family, friends, nobody, or others). The patients were further asked which of the symptoms they wished to be improved and what they thought could help them to improve their symptoms (open questions).

#### (iv) Further support

The patients were asked how easy or difficult it was to obtain support from GP, specialist, pharmacy, nursing service, family, friends or others. If the answer was “difficult”, the patients were asked to describe why it was difficult (open question). They were further asked whether they actively requested support (yes/no) and wished for more support (yes/no).

Demographic data were collected at the end of the survey.

### Data collection and analysis

During the first week after discharge, the interviewer (pharmacists) contacted the patients by telephone to make an appointment for the telephone interview. The interviewer wrote patients’ answers on a documentation sheet and documented the interview’s duration. For quality assurance, the interview was audio-recorded. Complete data were digitalized within 24 h of the interview. To ensure complete, accurate and comprehensible data, a second study person randomly checked every sixth data set for transcription errors and inconsistency. No irregularities were found. Data were analyzed descriptively. Open answers were categorized post hoc.

## Results

### Participants

**Fig. 1:**
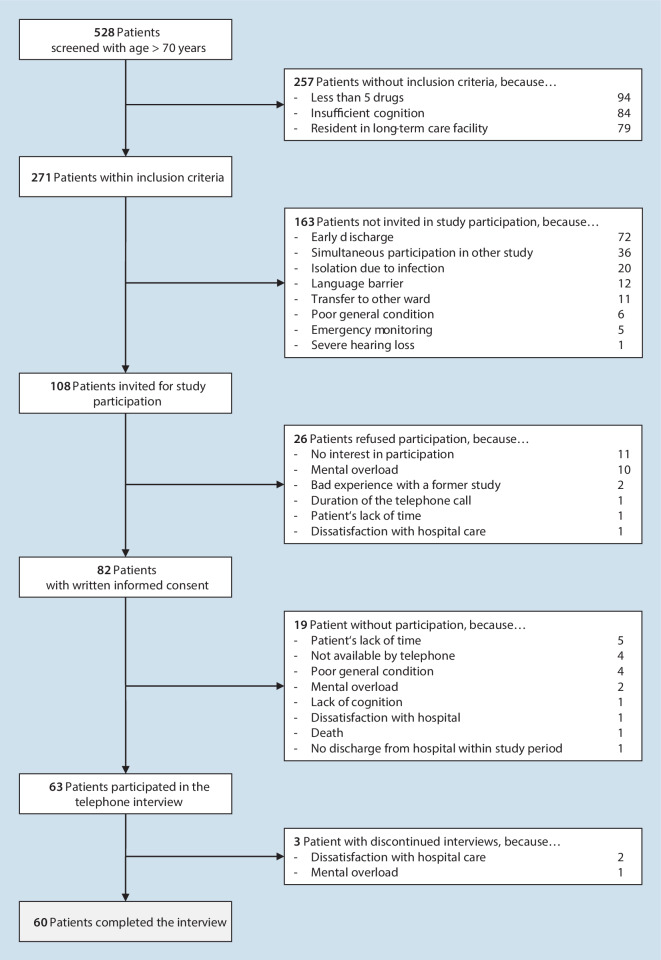
Flowchart of participants

A total of 60 patients (35 women and 25 men) completed the telephone interview (Fig. 1). We included 50 patients from the orthopedic wards and 10 from the rheumatological ward. In the median, the patients were 77 years old (min-max: 70–91 years) (Table 1). We performed the interview in median 12 days after discharge (Q25/Q75: 10/15 days; min–max: 5–37 days), which took 38 min (Q25/Q75: 31/50 min;min-max: 14–143 min).

**Table 1:** Patient characteristics (*N* total = 60)

Characteristics	Values
*Gender, n (%)*	–
Female	35 (58%)
Male	25 (42%)
*Age in years, median (Q25/Q75; min–max)*	77 (75/81; 70–91)
*Length of hospital stay in days, median (Q25/Q75; min–max)*	8 (5/10; 2–73)
*Inpatient department n (%)*	–
Orthopedic ward	50 (83%)
Rheumatological ward	10 (17%)
*Drugs at discharge, median (Q25/Q75; min–max)*	9 (7/11; 3–21)
*Postdischarge care*	–
Rehabilitation	12 (20%)
Nursing service	10 (17%)
*Place of residence, n (%)*	–
Metropolis (> 100,000 inhabitants)	34 (57%)
Medium-sized town (20,000–99,999 inhabitants)	6 (10%)
Small town (5000–19,999 inhabitants)	15 (25%)
Rural area (< 5000 inhabitants)	5 (8%)
*Highest level of education, n (%)*	–
Doctoral degree or higher	4 (7%)
University degree	24 (40%)
General qualification for university entrance	1 (2%)
Secondary school or comprehensive school certificate	30 (50%)
Without graduation	1 (2%)

*Q25* interquartile range 25%, *Q75* interquartile range 75%

#### (i) Symptom characteristics

**Fig. 2:**
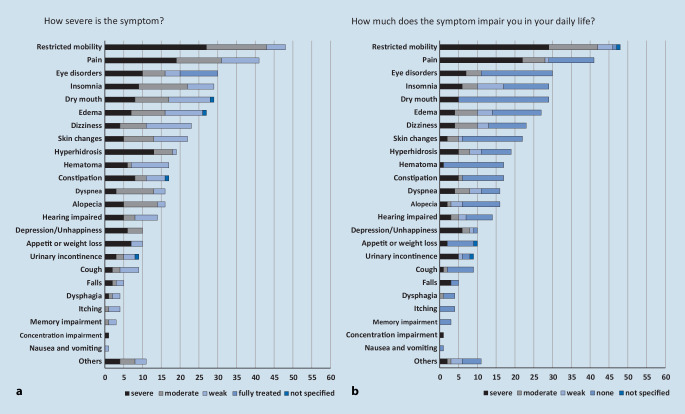
Symptom intensity (**a**) and impairment (**b**) from the patients’ perspective

**Fig. 3:**
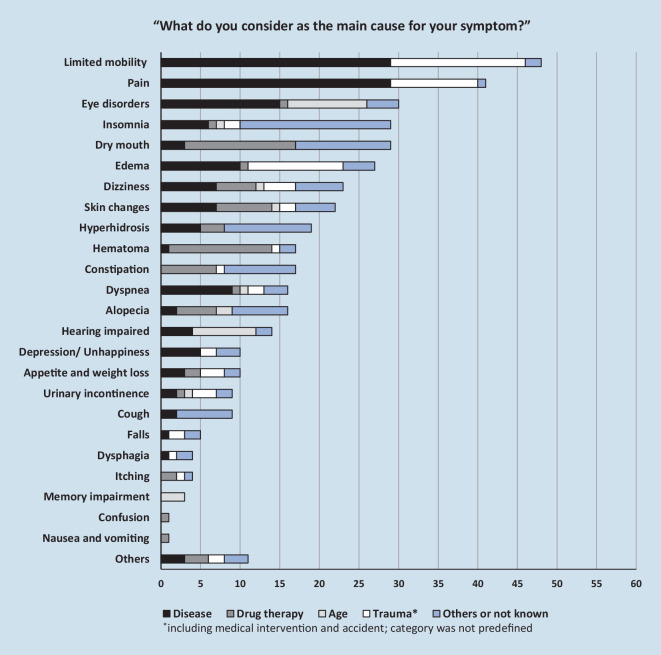
Self-assessed causes for the respective symptoms

The patients suffered from 6 symptoms in median (Q25/Q75: 5/8) with a range of 1 (min) to 14 (max) symptoms. Of all 415 symptoms reported by the participants, 155 (37%) were associated with severe intensity and 119 (29%) with severe impairment (Fig. 2). Of the patients 40 (67%) suffered from at least 1 severe impairing symptom. The patients already suffered from 356 (86%) symptoms since before their hospitalization, 30 (7%) symptoms first appeared during hospitalization and 28 (7%) after the patients’ discharge. In the latter case, dry mouth (4), alopecia (3), dizziness (3), edema (3), and skin changes (3) were most frequently named. Patients attributed the causes of 144 (35%) of their symptoms to the underlying disease, 68 (16%) to their drug treatment, 66 (16%) to experienced physical trauma, 28 (7%) to advanced age, and 109 (26%) to other causes (53, 13%) or not known (56, 13%, details in Fig. 3). For 43 of the 68 (63%) symptoms reported as being caused by drugs, patients were able to name a specific medication as the reason. Most frequently, they named anticoagulants (13, 19%), opioids (11, 16%) and co-analgesics (gabapentin, pregabalin, 8, 12%).

#### (ii) Symptom communication

The patients did not communicate 117 (28%) symptoms to a physician, a pharmacist, or family and friends. The most frequent symptoms that were not shared were dry mouth (23 patients), dizziness (10), insomnia (9) and hyperhidrosis (8). Although the interview guide did not ask why they did not communicate the symptom, some patients offered the reason unsolicited: “People already recognize the symptom without telling them”, “The pharmacy knows because of the prescriptions”, “There is no time for that during physician appointment”, “The physician has something more important to do”, “The symptom is normal in my age”, “Other patients also suffer from symptoms”, “You get used to it”, “I do not think it is alarming”, “I do not want to take more drugs”, “I am pleased about losing weight”, “People without the symptom do not understand it” and “Side effects of drugs must be accepted”.

#### (iii) Symptom treatment

**Fig. 4:**
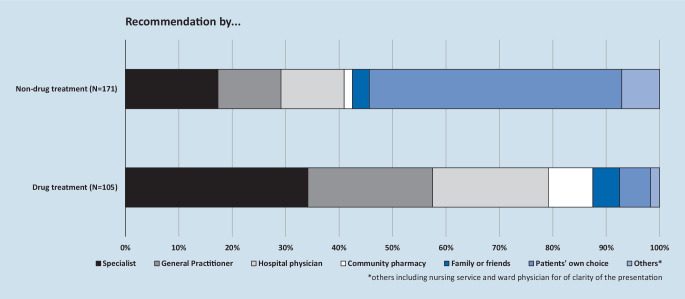
Recommendations for drug and nondrug treatment

A total of 280 symptoms were analyzed for measures the patients had already taken (the 5 most severe or impairing symptoms of each patient). Of these, 57 (20%) were treated only by drug therapy, 123 (44%) only by non-drug therapy and 48 (17%) by drug and non-drug therapy. Of these symptoms (19%) were not treated at all. Concerning the effectiveness, the patients considered 161 of their 228 symptom treatments (71%) as good, 41 (18%) as fair, 24 (11%) as poor, and 2 (1%) as not specified. Who recommended treatment options is shown in Fig. 4 and 52 (87%) of the participants wished for further improvement in at least 1 of their symptoms. Of the patients 24 (46%) who wished improvement of their symptoms reported specific suggestions, such as surgery, physiotherapy, a visit to a physician, rehabilitation, acupuncture, pain injection, electrotherapeutics, or exercise, 21 (40%) reported “wait and see” when asked what could help their symptoms to improve, 7 (13%) said “nothing can help”, and 7 (13%) said “do not know” (multiple categories possible).

#### (iv) Further support

**Fig. 5:**
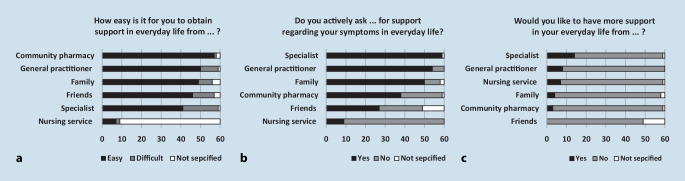
Patient support from interdisciplinary care: asking for support (**a**), easiness to obtain support (**b**), need for more support (**c**)

**Fig. 6:**
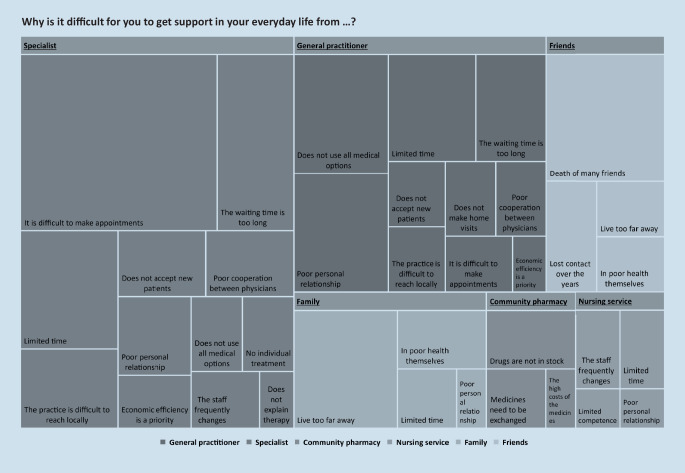
Causes for difficulties in getting support from healthcare professionals. Post hoc categories of the 125 patients’ answers to the open question. The size of the respective area correlates with the frequency of the category

Of the patients 59 (98%) stated that it was easy for them to obtain support, 60 (100%) of the patients actively asked for support, and 24 (40%) patients wanted to have more support (details in Fig. 5). The most often mentioned cause for any inability to obtain support was difficulties in making appointments with specialists (Fig. 6).

## Discussion

This telephone interview study analyzed older multimorbid orthopedic and rheumatic patients’ perspectives about their symptoms, symptom communication, treatment and further support after hospital discharge. The following findings could indicate gaps in symptom management: Firstly, the patients had not communicated almost every third symptom. Secondly, one fifth of the most impairing symptoms were not treated at all. Thirdly, most of the patients wished for further improvement in their symptom treatment. Surprisingly, the patients reported that it was relatively easy to obtain support. One can conclude that the interviewed patients reported remarkable, previously unnoticed symptoms despite their hospitalization and contact with physicians.

### Older orthopedic and rheumatic patients’ symptoms and the supposed causes of those symptoms

After their discharge from the hospital, the patients reported up to a maximum of 14 symptoms. Most of them already existed before hospitalization. Besides the expected symptoms of restricted mobility and pain, these patients were also affected by various other symptoms, such as insomnia or dry mouth. The fact that approximately one third of the symptoms were of severe intensity and impairment indicates a need for more effective treatment strategies, including deprescribing. For 16% of the reported symptoms, the patients considered drug treatment as the main cause, which might indicate adverse drug reactions. This is below the 28% rate reported in other studies [19]. Polypharmacy increases the risk of an adverse drug reaction, but nonspecific symptoms hamper its recognition [13]. Therefore, it can be assumed that the patients have considered only some of the possible existing drug causes for their symptoms.

### Lack of treatment due to insufficient patient communication

Although one third of the symptoms were not communicated by the patients before, they still desired improvement. “Wait and see” was a frequent answer regarding symptom treatment in our study, which is in line with the fact that older patients tend to wait or ignore their symptoms or manage them by themselves [1, 3]. Whitaker et al. provided examples for future interventions in symptom appraisal in cancer patients, which might be transferable to older multimorbid patients. They distinguished symptom knowledge, attention, expectation and identity [24]. Further measures are urgently needed to empower patients in self-management [12]. Measures should include the patients’ social and physical environment [5] as well as instrumental and emotional support [9].

### Implication for interprofessional symptom management

One fifth of the most alarming symptoms were not treated, which could indicate a gap in symptom management. Patients must first become aware of their physical changes and then discuss their symptoms with a healthcare professional [20]. The patients in our study seemed to lack sufficient motivation to communicate some of their symptoms. As the patients were interviewed after hospital discharge, however, they already had recent contact with physicians. They also considered it relatively easy to obtain support and actively ask for it. Therefore, it has to be questioned how these consultations can be followed by better overall symptom diagnosis and treatment. This treatment includes deprescribing, especially in the case of adverse events. It can be supposed that many symptoms were not addressed during inpatient treatment because of the complexity of older multimorbid patients. Ekdahl et al. generated a theory that a lack of time, competence and a holistic view hamper good care for older hospital patients [8]. For our respondents, pharmacies were the easiest to reach healthcare institutions but were less actively asked for support. This could be because the public is reluctant to trust pharmacy services [10]. Nevertheless, pharmacies counsel on self-medication to treat minor ailments and are interested in intensified interprofessional collaboration with other healthcare professionals [11]. It should be investigated how this low-threshold contact point can additionally support referring older patients to healthcare structures.

### Limitations

The number of 60 participants only allows limited conclusions about occasionally mentioned symptoms (limited generalizability). The nature of the interview as a telephone call resulted in the exclusion of patients with severe hearing problems. Our survey captured only the patients’ perspectives, which might differ from a professional assessment. The interview was quantitative; therefore, in-depth analysis could not be done. The interview guide was pretested but not validated. To reduce reporting and confirmation bias, we presented answers to all questions in the interview guide. Even though patients were asked to add further symptoms, it has to be considered that the number of 24 predefined symptoms influenced the number of reported symptoms. We included typical geriatric symptoms and frequent adverse events and only asked the patients questions regarding symptom treatment for the 5 most severe and impairing symptoms. We did not further differentiate between grades of clinical relevance. Although we consider deprescribing to be an equal treatment option, the interview did not specifically address this issue. Response bias, social desirability, and recall bias cannot be excluded.

## Practical conclusion

In the interview study after discharge, most of the older multimorbid orthopedic and rheumatic patients, who suffer from a variety of symptoms, had already experienced those symptoms before hospitalization. Patients considered their underlying disease as the predominant cause of their symptoms. A considerable number of symptoms had not been communicated or treated according to the patients, despite the patients having been hospitalized. Our findings underline the need for a higher awareness by healthcare professionals of the patient’s perspectives. A structured symptom assessment, as used in our interview study, could help to better capture the overall symptoms of older patients in routine care.

### Supplementary Information

Supplement 1: Translated questions of the interview guide and original German version

## Declarations

For this article no studies with human participants or animals were performed by any of the authors. The study has been approved by the appropriate ethics commitees. All studies mentioned were in accordance with the ethical standards indicated in each case.
