# Supplementary material for: Symptoms of older orthopedic and rheumatic patients: A telephone survey about symptoms, symptom communication, treatment and further support after hospital discharge
Source: Z Gerontol Geriatr. 2023 Jan 24;57(1):50–8. doi: 10.1007/s00391-022-02155-y (PMC10827833; doi:10.1007/s00391-022-02155-y)
Supplement: Supplementary file 1 — Supplement 1: Translated questions of the interview guide and original German version [file 391_2022_2155_MOESM1_ESM.pdf]

Supplement 1

Translated questions of the original German interview guide

The original questionnaire was conducted in German and can be found below. For a better understanding of the survey we translated the original questionnaire. Note that the following translation is not a professionally validated translation.

List of symptoms (follows the order of questioning): Skin changes, itching, alopecia, hyperhidrosis, restricted mobility, eye disorders, hearing impaired, dyspnea, dizziness, cough, dysphagia, dry mouth, nausea and vomiting, constipation, urinary incontinence, insomnia, memory impairment, depression/unhappiness, concentration impairment, pain, hematoma, edema, falls, appetite and weight loss, others.

1a) Which of the following symptoms do you currently experience? (category and wording of the explanation)

1b) When did the symptom first occur? (□ before hospitalisation, □ during hospitalisation, □ after discharge)

1c) How severe is the symptom? (□ fully treated, □ weak, □ moderate, □ severe)

1d) Does the symptom impair you in your daily life? (□ no, □ yes)

If yes: 1e) How much does the symptom impair you in your daily life? (□ weak, □ moderate, □ severe)

1f) What do you consider as the main cause for your symptom? (□ do not know, □ age, □ disease:______________, □ drug:______________, □ others:______________)

1g) With whom have you discussed the symptom before? (multiple categories possible, □ physician, □ pharmacy, □ others:__________________, □ nobody; notes to 1g:_________________)

For 5 most severe and impairing symptoms 1h) What measures do you take to improve the symptom?

Drug measures: (no, □ yes:_________________); Non-drug measures: (□ no, □ yes:_________________)

If 1h) „yes“: 1i) How effective have you found these measures? (□ good, □ fair, □ bad)

If 1h) „yes“: 1j) Who recommended the treatment? (multiple categories possible; □ GP, □ specialist, □ ward physician, □ community pharmacy, □ nursing service, □ family, □ friends, □ nobody (patient's own choice), □ others)

1k) Which of the symptoms do you wish to be improved? (________freetext_________)

1l) What do you think could help you to improve your symptoms? (________freetext_________)

List question 2: GP, specialist, community pharmacy, nursing service, family, friends, others

2a) How easy is it for you to obtain support in everyday life from ... ? (□ easy, □ difficult)

If 2a) „difficult“: 2b) Why is it difficult for you to get support in your everyday life from …? (________freetext_________)

2c) Do you actively ask… for support regarding your symptoms in everyday life? (□ yes, □ no)

2d) Would you like to have more support in your everyday life from…? (□ yes, □ no)

3) Sociodemographic data

How often do you visit your GP/specialist/community pharmacy? (at least 1x pro…□ 1 week, □ 1 month, □ 3 month, □ 6 month, □ 1 year, □ less than 1 year)

What is your highest level of education? (□ without graduation, □ secondary or comprehensive school, □ general qualification for university entrance, □ university degree, □ Doctor degree)

Collected from patient file: age, gender (□ male, □ female), Place of residence (□ metropolis, □ medium size city,□ small town, □ rural area); Number of drugs at discharge, length of hospital stay

Original German version of the interview guide

Symptomliste (Reihenfolge der Abfrage): Hautveränderungen, Juckreiz, Haarverlust, übermäßiges Schwitzen, Eingeschränkter Bewegungsmöglichkeit, Augenprobleme, Hörbeschwerden, Atemnot/ Kurzatmigkeit, Schwindel, Husten, Schluckprobleme, Mundtrockenheit, Übelkeit oder Erbrechen, Verstopfung, Inkontinenz, Schlafprobleme, Gedächtnisprobleme, Traurige Stimmung, Verwirrung, Schmerzen, Blutungen / Blaue Flecken, Ödeme /Schwellungen, Stürze, Appetit- oder Gewichtsverlust, Andere.

1a) Welches der genannten Symptome tritt zurzeit bei Ihnen auf? (Kategorie und Wortlaut der Beschreibung)

1b) Seit wann tritt das Symptom auf? (□ vor Krankenhaus, □ im Krankenhaus, □ nach Krankenhaus)

1c) Wie stark ist das Symptom? (□ vollständig therapiert, □ schwach, □ mittel, □ stark)

1d) Beeinträchtigt Sie das Symptom im Alltag? (□ nein, □ ja)

bei ja: 1e) Wie stark beeinträchtigt Sie dieses Symptom im Alltag? (□ schwach, □ mittel, □ stark)

1f) Was ist Ihrer Meinung nach der Hauptgrund für das Symptom? (□ weiß nicht, □ Alter, □ Erkrankung:______________, □ Arzneimittel:______________, □ Sonstiges:______________)

1g) Mit wem haben Sie bereits über das Symptom gesprochen? (Mehrfachauswahl möglich, □ Arzt, □ Apotheke, □ Sonstige:__________________, □ niemandem; Kommentar zu 1g:_________________)

Für 5 Stärkste und am stärksten belastende Symptome: 1h) Was tun Sie damit das Symptom besser wird?

Medikamentös: (□ nein, □ ja:_________________); nicht medikamentös: (□ nein, □ ja:_________________)

Bei 1h) „ja“: 1i) Wie gut helfen Ihnen diese Maßnahmen [jeweils]? (□ gut, □ mittel, □ schlecht)

Bei 1h) „ja“: 1j) Wer hat Ihnen diese Maßnahme [jeweils] empfohlen? (Mehrfachauswahl möglich; □ Hausarzt, □ Facharzt, □ Arzt im Krankenhaus, □ Apotheke, □ Pflegedienst, □ Familie, □ Freunde, □ Niemand, □ Sonstige)

1k) Von welchen Symptomen würden Sie sich wünschen, dass sie besser werden? (________Freitext_________)

1l) Was würde Ihnen darüber hinaus helfen, damit die Symptome besser werden? (________Freitext_________)

Liste Frage 2: Hausarzt, Facharzt, Apotheke, Pflegedienst, Familie, Freunde, Sonstige

2a) Wie leicht fällt es Ihnen sich im Alltag Unterstützung bei …einzuholen? (□ leicht, □ schwer)

Bei 2a) „schwer“: 2b) Warum fällt es Ihnen schwer, sich im Alltag Unterstützung bei ... einzuholen? (________Freitext_________)

2c) Holen Sie sich im Alltag aktiv Unterstützung bzgl. Ihrer Symptome bei …? (□ ja, □ nein)

2d) Würden Sie sich von …mehr Unterstützung im Alltag wünschen? (□ ja, □ nein)

3) Soziodemographische Daten

Wie oft gehen Sie zu Ihrem Hausarzt/Facharzt/Apotheke? (Mindestens 1x pro…□ Woche, □ Monat, □ Quartal, □ Halbjahr, □ Jahr, □ seltener)

Was ist Ihr höchster Bildungsabschluss? (□ keiner, □ Haupt-/Realschulabschluss, □ (Fach)Abitur, □ Studium, □ Promotion)

Aus Akte entnommen: Alter, Geschlecht* (□ männlich, □ weiblich), Wohnort* (□ Großstadt, □ Mittelstadt,□ Kleinstadt, □ ländliche Region), Anzahl Entlassmedikamente, Dauer des Krankenhausaufenthaltes
